# Maladaptive plasticity facilitates evolution of thermal tolerance during an experimental range shift

**DOI:** 10.1186/s12862-020-1589-7

**Published:** 2020-04-23

**Authors:** Aoife M. Leonard, Lesley T. Lancaster

**Affiliations:** School of Biological Sciences, Zoology Building, University of Aberdeen, Aberdeen, AB24 2TZ UK

**Keywords:** Countergradient variation, Maladaptive plasticity, Thermal fluctuations, Range-shifts, Genetic compensation, *Callosobruchus maculatus*

## Abstract

**Background:**

Many organisms are responding to climate change with dramatic range shifts, involving plastic and genetic changes to cope with novel climate regimes found at higher latitudes. Using experimental lineages of the seed beetle *Callosobruchus maculatus*, we simulated the initial phase of colonisation to progressively cooler and/or more variable conditions, to investigate how adaptation and phenotypic plasticity contribute to shifts in thermal tolerance during colonisation of novel climates*.*

**Results:**

We show that heat and cold tolerance rapidly evolve during the initial stages of adaptation to progressively cooler and more variable climates. The evolved shift in cold tolerance is, however, associated with maladaptive plasticity under the novel conditions, resulting in a pattern of countergradient variation between the ancestral and novel, fluctuating thermal environment. In contrast, lineages exposed to progressively cooler, but constant, temperatures over several generations expressed only beneficial plasticity in cold tolerances and no evolved response.

**Conclusions:**

We propose that thermal adaptation during a range expansion to novel, more variable climates found at high latitudes and elevations may typically involve genetic compensation arising from maladaptive plasticity in the initial stages of adaptation, and that this form of (countergradient) thermal adaptation may represent an opportunity for more rapid and labile evolutionary change in thermal tolerances than via classic genetic assimilation models for thermal tolerance evolution (i.e., selection on existing reaction norms). Moreover, countergradient variation in thermal tolerances may typically mask cryptic genetic variability for these traits, resulting in apparent evolutionary stasis in thermal traits.

## Background

In response to climate change, many species are range shifting to take advantage of new colonisation opportunities in habitats located pole-wards of their ancestral range, where temperatures have recently warmed to surpass minimally-suitable conditions for population growth [27]. This opportunity is allowing many species to exhibit larger range sizes than in previous centuries [23]. However, in comparison to ancestral range limits, these higher latitudes also exhibit greater daily and annual thermal variation and lower temperature minima, which are not necessarily ameliorated by warming trends [30, 31, 48]. Mean monthly land surface temperatures vary less than 5 °C at 0° but over 20 °C at 60° latitude [31, 56]. Furthermore, high latitude climates are more likely than lower latitudes to be characterised by occasional extreme weather events such as severe storms and cold snaps that will impose infrequent but extreme selective pressure on the range-shifting populations [1, 24, 56, 61]. Thus, during range expansions facilitated by warming climates, organisms face novel climatic selection pressures in the new part of the range [36], despite the fact that range shifts typically track mean suitable climates [7]. Successful range shifts therefore often require adaptations to these more extreme cold events and more variable temperatures ([2, 8, 10, 30, 37]; AL and LL, unpublished results).

One well-studied consequence of latitudinal variation in environmental temperature is that populations at higher latitudes tend to exhibit broader thermal tolerances than lower-latitude populations, putatively in order to cope with greater climatic variability there (The Climate Variability Hypothesis [1, 45, 63], but see [35] for an alternative hypothesis). However, experimental evidence for the influence of environmental thermal variability on the evolution of thermal tolerances remains equivocal: some previous research has suggested that high levels of environmental variability may limit the expression of beneficial thermal acclimation, even in cases where the environmental temperature fluctuations are regular and predictable [6, 59]. Moreover, the physiological stress of fluctuating temperatures is often inferred to have deleterious developmental effects that can negatively impact adaptive responses to changing mean temperatures by reducing both the optimal and the critical maximum temperature [47]. Despite these potentially common intrinsic costs of developing under variable conditions, other studies have suggested that adaptation to temperature fluctuations may actually be beneficial for the development of adaptive thermal tolerances [5] and fitness [4]. Fluctuating temperatures can also potentially mitigate prolonged temperature stress which may otherwise be lethal, by allowing physiological or behavioural preparation for subsequent extreme thermal exposures [43].

In cases where the range limit environment is more variable than the ancestral environment, and thus stressful enough to invoke maladaptive plasticity (i.e., an environmental effect on the phenotype which moves fitness further away from the optimum in comparison to that which is expressed by a more canalised phenotype [18]), range-edge populations may evolve novel genetic changes to counteract the deleterious or suboptimal phenotypic effects of their new environment [20]. Under this process of genetic compensation, the mean trait values of core and range edge populations may be more similar in the field than when reared in a common environment, because selection results in evolutionary changes that serve to re-establish the phenotype which is favoured in both the new and the ancestral environments [20]. This process can result in a latitudinal cline in genetic contributions to traits known as counter-gradient genetic variation, whereby latitudinal variation in the genetic contribution to a trait exhibits the opposite trend from latitudinal variation in the environmental effect on the expressed phenotype [14, 20, 34]. Genetic compensation and the pattern of counter-gradient genetic variation that it produces underlie a cryptic form of local adaptation, because these evolutionary processes arise in response to strongly spatially divergent patterns of directional selection, but act to make populations in different environments more similar to one another. Such a negative covariance between environmental and genetic influences may give a superficial impression of lack of local adaptation, and conceal even substantial genetic differentiation along spatial gradients [40].

Despite the fact the deleterious effects of thermal environments on thermal tolerances have previously been reported (see above), genetic compensation and counter-gradient variation in thermal tolerances have seldom been investigated, likely because many studies of thermal plasticity focus on beneficial acclimation effects [22, 39, 52]. There is thus a major research gap in our understanding of how maladaptive thermal plasticity influences the process of thermal tolerance evolution. In order to address this research gap, we conducted experimental evolution studies simulating a geographic range shift to 1) cooler, or 2) cooler and more variable temperatures, and observed the resulting (improved or worsened) phenotypic responses to thermal stressors (in the form of acute thermal tolerances, CT_min_ and CT_max_; [28, 44, 60]. We further assessed the contribution of evolved, developmental, and short-term acclimation effects to observed thermal tolerances expressed by individuals from each of the evolved treatments. By decomposing these influences, we aimed to understand the conditions under which the direction of evolutionary change in thermal tolerances aligns with or opposes the effects of phenotypic plasticity, in the early stages of adaptation to progressively novel climates. We predicted that (i) During the initial stages of adaptation to progressively colder temperatures, maladaptive plasticity may be more pronounced when newly-encountered temperatures are also increasingly variable (rather than constant) within generations, as populations must cope with two axes of novel environmental variability (changes in environmental mean and variance). (ii) Evolved changes in thermal tolerance may be more rapid in the presence of maladaptive plasticity (the genetic compensation hypothesis), than when the thermal environment has beneficial effects on thermal tolerance (the genetic assimilation hypothesis; whereby environmentally induced phenotypic variation no longer requires the environmental signal to be expressed [50]). Addressing these hypotheses will allow us to understand how plasticity and evolved changes contribute to novel thermal tolerances during progressively cooler and more variable conditions, such as often experienced during pole-ward range shifts.

## Methods

### Source population

We use as our model system experimental lines obtained from a tropical (i.e., pre-range expansion) population of an expanding global crop pest, *Callosobruchus maculatus. C. maculatus* is a globally relevant crop pest spreading pole-ward under current global warming, facilitated by the global legume trade [62]. The *C. maculatus* beetles used in this experiment were sourced from Niamey, Niger and have been maintained in an outbred laboratory population on a diet of cowpeas, *V. unguicularis*, at a constant 27 °C thermal regime and 35% relative humidity with a 12:12 h light/dark photoperiod for 19 years or around 300 generations ([15, 51]; Paul Eady personal communication). Given the region of ancestry and long-term rearing conditions, our beetles are therefore likely well adapted to these constant, narrow thermal conditions.

### Establishment of experimental lines

**Fig. 1:**
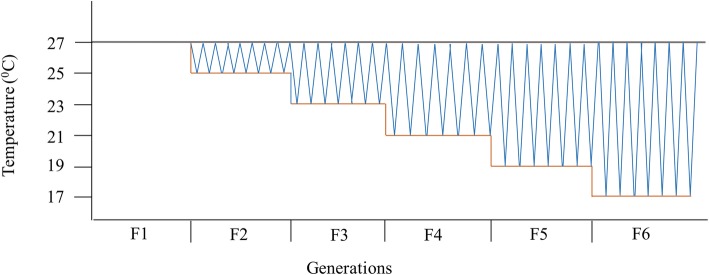
Schematic drawing of evolved lines. The fluctuation line was exposed to increasingly cooler and more fluctuating, daily temperatures, maintaining the upper thermal limit but reducing the lower thermal limit by 2 °C each generation until the sixth generation experienced a temperature fluctuation between 17 and 27 °C per day (blue). The constant-decline evolved line was reared such that each generation experienced a constant temperature, but this temperature was decreased by 2 °C at the start of each new generation (red). Control conditions were maintained at a constant 27 °C over all generations (grey). Depicted frequency of temperature fluctuations is not to scale

Our experimental design included two experimental evolution treatments and a control treatment, each with 5 replicate lines (Fig. 1). Each generation was formed by adding ca. 1000 newly emerged adults, estimated by volume (3 ml), to a jar containing ca. 100 g of dried black eyed beans. This corresponds to moderate larval competition under ancestral conditions [51]. The fluctuating treatment lines were evolved under novel, increasingly more fluctuating, daily temperatures. For this we maintained the upper daily thermal limit at the ancestral 27 °C, but reduced the lower daily thermal limit by 2 °C each generation (where each generation lasts approximately 21 days), until the sixth generation experienced a daily fluctuation between 17 and 27 °C on a daily temperature cycle. A 2 °C daily temperature cycle was programmed such that within each 24 h period, beetles experienced 4 h of ramping from 17 °C to 27 °C, 8 h at 27 °C, 4 h ramping from 27 °C to 17 °C, and 8 h at 17 °C (one complete thermal cycle per day). I.e., generation 1 experienced a constant 27 °C, whereas generation 2 experienced temperatures that fluctuated between 25 °C and 27 °C. Generation 3 experienced daily fluctuations between 23 °C and 27 °C, etc. (blue line, Fig. 1). The constant decline treatment consisted of five lines exposed to a 2 °C per generation decline in constant temperature, with no fluctuation regime (red line, Fig. 1). This treatment allows populations to adjust to the same progressively novel, minimum temperature conditions as the fluctuating treatment, but in the absence of fluctuations. The control treatment lines were maintained at 27 °C throughout the experiment (grey line, Fig. 1). All lines were housed in programmable cooling incubators (LMS model 280NP refrigerated incubator; Kent, UK) throughout the experiment in a 12:12 daily light cycle. A 2 °C decrease in temperature per generation was chosen as this represents a common daily fluctuation at the equator, which then increases to 8–10 °C daily fluctuations at 30° latitude [65] (in comparison to 10 °C daily fluctuations in our treatment). While the speed of pests spread varies drastically [3], a 10 °C change in the climate gradient over which a poleward pest invasion occurs is not unlikely in as little as 6 generations.

Prior to the experiment, we conducted pilot studies which suggested that each female experiences a moderate decline in fitness under reduced temperature treatments. Fecundity is reduced from 67.9 ± 2.83 SE adult offspring being produced per female (*n* = 20) at 27 °C, to 47.5 ± 2.93 adult offspring being produced per female (n = 20) at 23 °C (ancestral temperature - 4 °C). Thus our evolution treatments of 2 °C shifts per generation are sufficient to produce significant selection pressure on individuals without invoking bottleneck effects. Our pilot studies further indicate a CT_min_ of 19 °C and a CT_max_ of 39 °C for fitness (offspring emergence rates), using females from the ancestral (27 °C adapted) laboratory population (AL and LL, unpublished results), indicating that the endpoint of our experimental treatments (17 °C) is outside the range of ancestrally tolerable temperatures, and allowing us to conclude that evolved changes have occurred*.* However, the fact that our control lines cannot survive at the evolved temperature regime limits our ability to compare derived and ancestral plasticities under these conditions (see below).

**Fig. 2:**
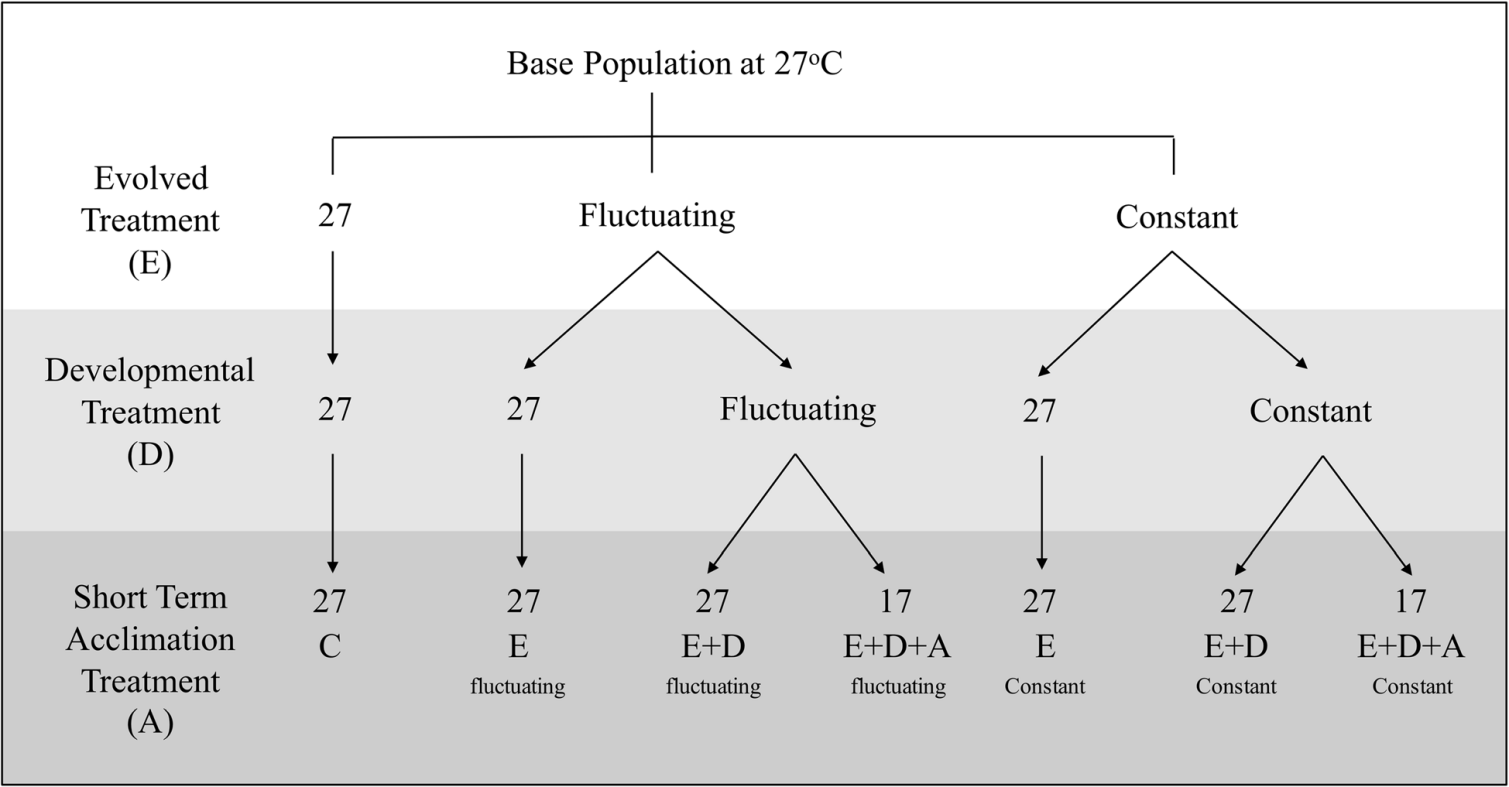
Experimental design of the experiment. Evolved lines (fluctuating or constant thermal decline over 6 generations; *n* = 5 lines per treatment) were split into one of two developmental treatments, in which individuals from each replicate line were reared under their evolved conditions, or were reverted back to their ancestral conditions (27 °C) (E). Individuals from lines which were maintained in their evolved conditions for the developmental treatment were then exposed to either a short-term acclimation treatment of 17 °C, representing the lower limits of their treatments (E + D + A), or were acclimated under ancestral conditions (27 °C) (E + D) prior to the start of thermal trials. A constant treatment (also with n = 5 lines) was maintained at 27 °C throughout. Each of the evolved and developmental treatments is represented by n = 5 replicate lines. The acclimation treatments are represented by *n* = 40 individuals total per developmental treatment category

At the end of generation 6, we assessed evolved and plastic responses to temperature in each of the treatment and control lines. To do this, individuals emerging as adults from each line were split into 2 developmental treatments representing the ancestral (27 °C) and evolved thermal conditions (see Fig. 2 for a schematic depiction of these conditions), with *n* = 1000–1500 individuals from each replicate of each line allocated to each developmental treatment. For those lines developing under novel conditions (represented by the red and blue lines, respectively, in Fig. 1), we further imposed either an ancestral or evolved acclimation treatment (*n* = 40 individuals per line per acclimation treatment). Both control and evolved individuals that were reared under common garden conditions at 27 °C were also only acclimated at 27 °C. Developmental and acclimation treatments were designed to disentangle effects of evolved changes in thermal tolerances, effects of developmental plasticity, and effects of acclimation temperatures on thermal tolerances. The resulting evolved/developmental/acclimation treatments therefore consisted of: 1) an ‘evolution only’ (E) treatment in which individuals were evolved to a novel (constant or fluctuating) thermal regime, but the final generation was reared for one complete generation in a common garden environment of a constant, ancestral 27 °C, and also were not provided any short-term acclimation opportunity in the new thermal regime, 2) an ‘evolution and developmental plasticity’ treatment (E + D) in which individuals were evolved to a novel thermal regime, and the final generation was also maintained under the conditions to which they had most recently evolved (i.e., not transferred to the common garden for 1 generation); however the final generation individuals were transferred to 27 °C for 30 min prior to the thermal tolerance assessments, to deprive them of short term acclimation benefits in the novel, evolved thermal conditions and 3) an ‘evolution, developmental plasticity, and acclimation’ treatment (E + D + A) in which individuals were evolved under their respective novel regimes, the final generation was also reared under the ancestral regime, and test individuals were also acclimated to one 30 min acclimation period to the novel, cooler portion of the temperature range (17 °C) prior to thermal tolerance assessment. A short acclimation time of 30 min typically produces a ‘rapid hardening’ response, which is well described in insects, and is mechanistically distinct from longer-term, developmental acclimation processes [41]. Our design allows us to disentangle these two forms of thermal plasticity from each other and from evolved changes in thermal tolerance. The control lines were evolved, reared, and acclimated under ancestral, constant conditions (C), and we did not test thermal plasticity of these lines as the unevolved control lines are unable to survive and reproduce at the experimental temperatures (17 °C) (Fig. 2). Individuals from E, E + D, and E + D + A treatments were each balanced over the 5 replicate lines within each evolved conditions (fluctuating or constant), with 8 individuals per replicate assessed for heat or cold tolerances (8 individuals * 5 replicates * 7 treatments * 2 thermal tolerance tests [heat/cold] = 560 individuals tested for thermal tolerances overall). See Fig. 2 for a schematic representation of our design.

### Thermal tolerance assays

We employed a ‘reaction norm’ approach to phenotypic plasticity which assess variation in mean trait values across distinct environments, in which critical thermal limits represent threshold traits corresponding to continuous, underlying physiological processes. We defined critical thermal limits as the ability to maintain motor function at a particular temperature [9, 46, 55]. Although acute thermal limits may not be linked to variation in thermally-dependent reproductive rates, thermal physiological limits can predict survivorship under extreme weather events that are likely to cause high mortality and local extinctions, and which can thus limit geographic ranges.

Thermal trials were conducted by placing newly-emerged adult beetles into individual chambers (2 ml Eppendorf tubes with cotton wool pushed half way down to prevent escape but promote ventilation). The chambers were then floated in a programmable water bath (Grant model TC120-R4) such that the beetle and its air space were below the surface of the water. An external probe was used to monitor temperature in the airspace and adjust the bath temperature to maintain the airspace at the desired (programmed) temperature. The probe also assessed differences between the programmed and actual temperatures in the air space; these were always < 0.1 °C. For the heat tolerance trials, starting from the temperature at which individuals were short-term acclimated (27 °C or 17 °C), environmental temperatures were increased at a rate of 0.1 °C min^− 1^ until beetles’ loss of muscle function occurred, and at that point the critical thermal maximum (CT_max_) was assessed for each individual [28, 44, 60]. Cold tolerance trials were similar except that temperatures were decreased instead of increased over the trial.

### Statistical analyses

Differences in heat and cold tolerances between the four treatments (C, E, E + D, E + D + A) were compared separately for fluctuating and evolved lineages, using linear models in base R [57], and using type I sums of squares in which each unique treatment (comprising a distinct combination of evolved, developmental, and acclimation conditions) is compared to the control treatment. Separate models were run for heat- and cold-ramping assays, and a Gaussian error distribution was fit to the data. Both heat and cold tolerance were approximately normally distributed, and visual examination of model residuals revealed that this error distribution was appropriate. Both treatment and sex were initially included as fixed effects, but sex did not have a significant effect on thermal tolerances, and did not improve AIC of model fit, so it was dropped from final reported models. Evolution, developmental and acclimation regimes could not be tested as independent factors (i.e., in a crossed model where interactions between E * D * A levels of treatment could be tested), because developmental treatments are in fact nested within evolved treatments. However, nested models also cannot be applied to our data because developmental treatments could not be applied in controls (with 100% mortality of control individuals reared at evolved temperatures, methods section). Thus fitting each unique *combination* of evolved, developmental, and acclimation treatments as a separate experimental condition represented the best way to model the data, with pairwise tests used to assess how each E, D, or A treatment resulted in altered thermal tolerances. Thus we compared each treatment to baseline controls, and also conducted post hoc comparisons between E, E + D and E + D + A treatments, applying a Bonferroni correction for multiple comparisons with ∝ = 0.0167 (Additional file 1: Table S2). We also conducted analyses to disentangle potential effects of experimental replicate line on resulting thermal tolerances (see Additional file 1: Table S1) —this effect did not qualitatively impact main effects of treatment on thermal tolerances.

## Results

### Recent adaptation to cooler, fluctuating temperatures

**Fig. 3:**
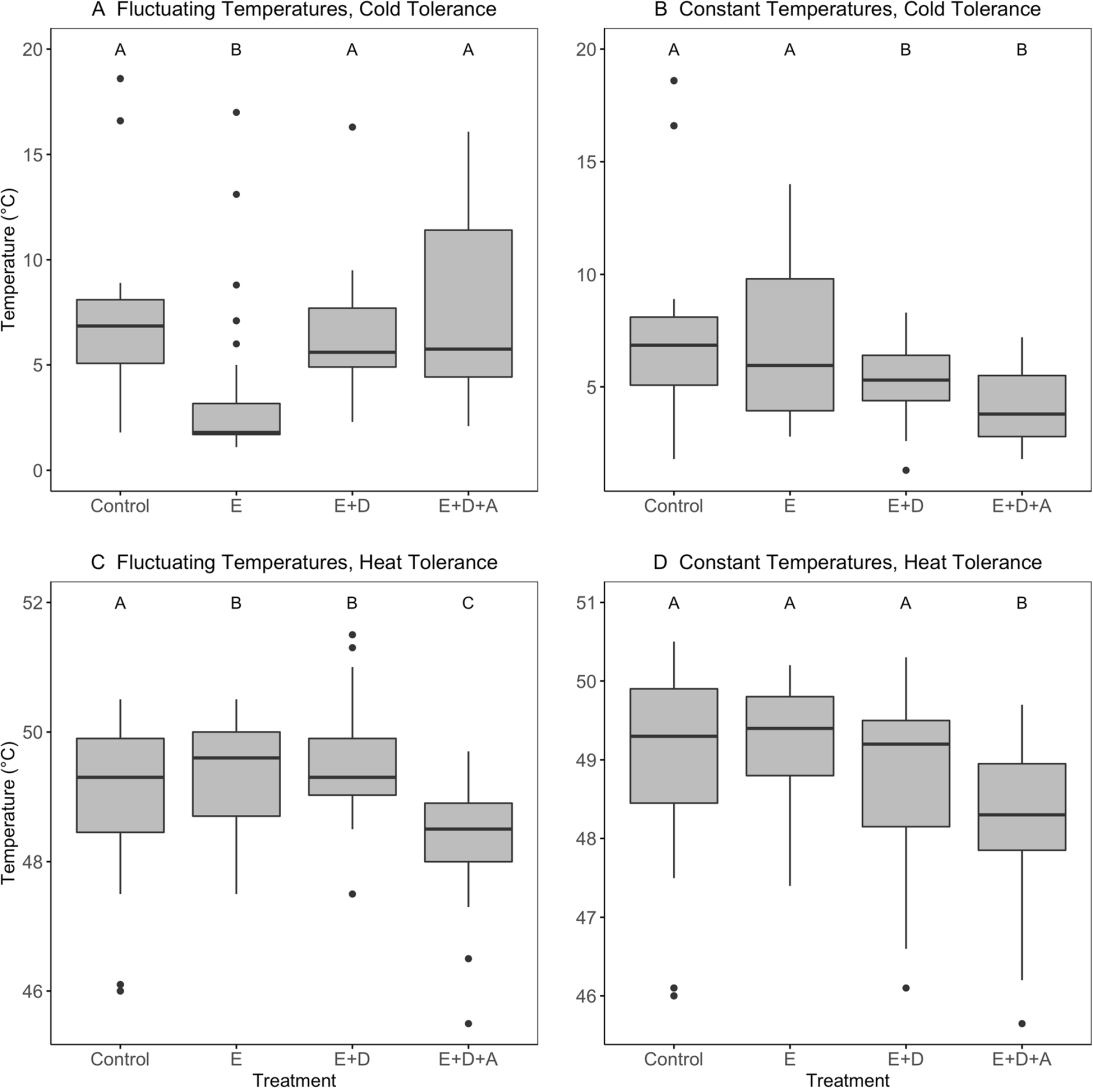
**A**,**C**: Effect of experimental shifts to cooler, more variable temperatures on cold thermal tolerance (**A**) and Heat tolerance (**C**). **B**,**D**: Experimental shifts to cooler, constant temperatures on cold thermal tolerance (**B**) and Heat tolerance (**D**). See *Establishment of Experimental Lines* for details of treatment categories. Letters correspond to statistically significant differences between treatments, where shared letters denote no significant differences between treatments

We found that cold tolerance was improved in comparison to controls following adaptation to colder, fluctuating temperatures, but only when individuals were reared for one generation under ancestral conditions (constant 27 °C) (effect of E_fluctuating_ treatment on CT_min_ = − 3.98 ± 0.8 SE, t_3,156_ = − 4.973, *p* = 1.72E-06). However, this evolved benefit is not observed when individuals are reared in the fluctuating thermal environment in which they had recently evolved (effect of E + D_fluctuating_ treatment on CT_min_ = − 0.77 ± 0.8 SE, t_3,156_ = − 0.956, *p* > 0.05), nor when they are also acclimated to this temperature (effect of E + D + A_fluctuating_ treatment on CT_min_ = 0.33 ± 0.8 SE, t_3,156_ = 0.413, *p* > 0.05) (Table 1; Fig. 3). This suggests that evolved changes in cold tolerances have arisen to counteract significant maladaptive plasticity imposed by the developmental environment, and that the evolved benefits are therefore only observable in the ancestral environment (consistent with the genetic compensation hypothesis). Concomitantly, cold tolerance of E + D_fluctuating_ lines is worse compared to E_fluctuating_ cold tolerance (CT_min_ = 3.2150 ± 0.7815 SE, t_3,156_ = 4.114, *p* = 7.27e-05). However, there is no additional effect of short term acclimation on cold tolerance (E + D + A_fluctuating_ compared to E + D_fluctuating_; CT_min_ = 1.0958 ± 0.7815 SE, t_3,156_ = 1.402, *p* = 0.164, Additional file 1: Table S2), suggesting that maladaptive plasticity in cold tolerance results primarily from developmental, rather than short-term, influences.

**Table 1:** Effect of evolved, developmental, and short term acclimation effects on both cold and heat tolerance of recently cold-adapted individuals, in comparison to ancestral thermal tolerances. See Additional file 1: Table S2 for post-hoc comparisons of E vs. E + D and E + D vs. E + D + A effects on thermal tolerances

Lines	Treatment	Cold Tolerance	Estimate	SE	Pr(>|t|)	Heat Tolerance	Estimate	SE	Pr(>|t|)
Control	C	Baseline				Baseline			
Fluctuating Decline	E	Improved	−3.98	0.8	1.72E-06	Improved	0.42	0.214	0.04
	E + D	Same	−0.77	0.8	0.05	Improved	0.57	0.214	0.008
	E + D + A	Same	0.33	0.8	0.05	Worse	−0.59	0.214	0.006
Constant Decline	E	Same	−0.09	0.64	0.8	Same	0.31	0.23	0.17
	E + D	Improved	−2.00	0.64	0.002	Same	−0.095	0.23	0.6
	E + D + A	Improved	−2.98	0.64	7.6e-06	Worse	−0.56	0.23	0.0151

Adaptation to colder, fluctuating temperatures also confers an evolved benefit of increased heat tolerance in comparison to controls, despite the upper environmental temperature remaining unchanged over the course of the experiment. Like the evolved change in cold tolerance, this adaptive change in heat tolerance is observed in evolved individuals that were reared at the ancestral (constant 27 °C) temperature (effect of E_fluctuating_ treatment on CT_max_ = 0.42 ± 0.214 SE, t_3,156_ = 1.99, *p* = 0.04), but unlike cold tolerance, we also see that the improved heat tolerance is retained by individuals when reared under their evolved thermal regime (effect of E + D_fluctuating_ treatment on CT_max_ = 0.57 ± 0.214 SE, t_3,156_ = 2.67, *p* = 0.008; E + D_fluctuating_) compared to E_fluctuating_, *p* = 0.437, Additional file 1: Table S2). However, short-term acclimation to cooler temperatures within the novel, evolved thermal range negatively impacts the heat tolerance of evolved lineages, reducing it to below that exhibited by control, E, or E + D individuals (effect of E + D + A_fluctuating_ treatment on CT_max_ = − 0.59 ± 0.214 SE, t_3,156_ = − 2.776, *p* = 0.006; see Additional file 1: Table S2 for additional pairwise comparisons). Thus, overall, we see that recent adaptation to cooler and more variable temperatures results in evolved improvements in both heat and cold tolerance, but these improvements are reversed under different developmental and short-term acclimation scenarios within the range of conditions to which the populations have recently adapted.

### Recent adaptation to cooler, constant temperatures

In contrast to the fluctuating lines, adaptation to a constant decline of 2 °C each generation does not result in any clear evolved effects on cold tolerance, despite the fact that the lines evolved under the constant decline conditions reached the same thermal minimum as lines evolved under the fluctuating regime (17 °C). When reared in a common garden (27 °C), there was no difference between evolved and control individuals in their lower thermal tolerances (effect of E_constant_ treatment on CT_min_ = − 0.09 ± 0.64, t_3,156_ = − 0.15, *p* = 0.8). However, both short- and long-term acclimation to cooler temperatures improved the cold tolerances of these individuals in comparison to control individuals: (effect of E + D_constant_ treatment on CT_min_ = − 2.00 ± 0.64, t_3,156_ = − 3.11, *p* = 0.002; effect of E + D + A_constant_ treatment on CT_min_ = − 2.98 ± 0.64, t_3,156_ = − 4.63, *p* = 7.6e-06). Moreover, for E + D_constant_, there was a beneficial increase in cold tolerance in comparison to E_constant_ (effect = − 1.9075 ± 0.5565 SE, t_3,156_ = 3.428, *p* = 0.00084; Additional file 1: Table S2). However, there was no difference in cold tolerance between E + D + A_constant_ to E + D_constant_ (CT_min_ = − 0.9800 ± 0.5565 SE, t_3,156_ = − 1.761, *p* = 0.08083; Additional file 1: Table S2), suggesting that observed beneficial plasticity was developmental in origin. Thus, in contrast to the fluctuating treatments, we observe no evolutionary change in thermal tolerances under constant, evolved thermal declines. Instead, we observe adaptive plasticity in response to (constant) developmental temperatures, and we observe no evidence of cold stress in response to either developmental or short-term exposure to the novel, colder environment (no evidence for maladaptive plasticity in cold tolerances under constant temperature decreases).

Similarly, adaptation to constantly declining, cooler temperatures resulted in no evolutionary change in heat tolerances in comparison to controls (effect of E_constant_ treatment on CT_max_ = 0.31 ± 0.23, t_3,156_ = 1.36, *p* = 0.17). Developmental exposure of the evolved lines to their evolved thermal regime also had no impact on heat tolerance, as long as individuals were subjected to short-term (30 min) acclimation to the ancestral 27 °C temperature (effect of E + D_constant_ treatment on CT_max_ = − 0.095 ± 0.23, t_3,156_ = − 0.42, *p* = 0.6; there was also no difference in heat tolerance between E + D_constant_ compared to E_constant_; see Additional file 1: Table S2). However, in the absence of any opportunity of individuals from E + D lines to acclimate to the ancestral temperature regime, heat tolerance is reduced (effect of E + D + A_constant_ treatment compared to controls on CT_max_ = − 0.56 ± 0.23, t _3156_ = − 2.45, *p* = 0.0151; effect of E + D + A_constant_ compared to E + D_constant_; CT_max_ = − 0.4662 ± 0.2072 SE, t_3,156_ = − 2.250, *p* = 0.0263; Additional file 1: Table S2). Thus in both fluctuating-decline and constant-decline evolved treatments, short-term exposure to low temperatures reduces heat tolerances, whereas developmental acclimation to these temperatures does not. Only the fluctuating-decline treatment, and not the constant-decline treatment, resulted in evolved changes in heat tolerance.

In short, we find that adaptation to cooler, constant temperatures does not result in maladaptive plasticity, nor does it result in evolved changes in heat or cold tolerance. In contrast, adaptation to cooler, fluctuating environments results in patterns of maladaptive cold tolerance plasticity (although some adaptive plasticity in heat tolerances was observed). This maladaptive plasticity in cold tolerances is associated with beneficial, evolved changes in both heat and cold tolerance. However, the combined effect of evolved benefits and maladaptive plasticity in the novel, fluctuating environment produces a pattern of countergradient variation, such that the expression of the evolved benefit is not observed in the novel (evolved) environment.

## Discussion

In this study we compared evolved thermal tolerances under various conditions of short- and long-term acclimation to the novel and ancestral environment, during the first six generations of adaptation to a) progressively cooler or b) progressively cooler and more variable temperatures, such as might be experienced by populations during the initial stages of a range shift to higher latitudes or elevations, which are characterised by both cooler and often more variable climates. We find that adaptation to novel climates that are both cooler and more variable in comparison to ancestral climates results in rapid evolutionary change in thermal tolerances, producing adaptive shifts in both heat tolerance and cold tolerance in comparison to the ancestral population. However, these benefits are only realised when individuals adapted to the novel climate are reared or acclimated in the common garden representing the ancestral climate regime. This suggests that recently evolved responses to novel temperature regimes under fluctuating conditions are consistent with the pattern of countergradient variation, in which adaptation to harsher conditions only results in observable phenotypic change when observed under a benign, common-garden environment [14]. The results also indicate that maladaptive plasticity in cold tolerance may be a common feature of the initial stages of recent adaptation to novel climates depending on how pronounced the stress is. The pattern has been well described in other traits such as growth rate, developmental rates, and thermoregulatory behaviour [12–14, 40, 43, 54], however countergradient variation in thermal tolerances is seldom reported (i.e., higher heat tolerances exhibited by individuals from populations adapted to cold climates than warm-adapted populations, when reared under common-garden conditions; e.g., [19, 54]). If common in nature, such a pattern may help explain why thermal tolerances are often observed to exhibit strong geographical stasis [17, 21, 26].

In contrast, lines adapted to novel, cooler, constant conditions did not exhibit evolved changes in heat or cold tolerance, and did not exhibit maladaptive thermal plasticity (but did exhibit beneficial plasticity in cold tolerance, which may be ancestral or derived). We therefore find no evidence here for a genetic assimilation-like process by which selection acts on thermal plasticity reaction norms [39]. This result is linked to the idea that plasticity can impede evolutionary change when it acts in the same direction as selection [25]. Under fluctuating thermal regimes, in contrast, we find that cold tolerance plasticity acts in the opposite direction of selection, a phenomenon which is well known to accelerate the rate of adaptive evolution [18, 53]. Many previous studies have examined differences in adaptive responses to constant vs. fluctuating environments [5, 11, 42, 47, 53, 59], but this is, to our knowledge, the first study indicating that thermal adaptation depends on the presence of temperature fluctuations in the novel environment in order to occur. Overall, this suggests that knowledge of a population’s evolutionary history, in terms of the recent and ongoing climates to which it has adapted or is adapting, is important in interpreting environmental tolerances when assessed either in the wild or in the laboratory [35]. Moreover, caution must be used when inferring evolved changes in thermal tolerances as this may in part actually represent maternal effects, particularly when a parents’ rearing environment is also likely a strongly selective environment. Further, the reported short-term acclimation effects on heat tolerance may also be due to a number of factors including both starting temperature of the trial and the duration of the thermal trial (which necessarily results when the short term acclimation temperature is lower, necessitating lower starting temperatures for the thermal trials [58]). Our results suggest that thermal adaptation via selection on existing, beneficial thermal reaction norms takes significantly longer evolutionary time than the time frame required for genetic compensation due to maladaptive plasticity, which we observed in this experiment over 6 generations.

Our pilot studies indicate that control lines of *C. maculatus* are unable to achieve reproductive success at 17 °C, however both our fluctuating and constant evolved lines survived and reproduced well at this temperature (personal observation), despite the fact that neither of our evolved lines exhibited evolved changes in thermal tolerance which were beneficial in the new environment. This experimental outcome supports a growing body of work suggesting that absolute thermal tolerances may typically be evolutionary decoupled not only from each other, but also from the evolution of performance traits within the absolute tolerance range [26, 33, 59]. Our study did not directly address the multivariate nature of adaptation to novel constant or fluctuating thermal conditions. However, any recently-evolved phenotype is necessarily influenced by the genetic variances and covariances of important evolved and ancestral traits [49]. An unfavorable correlation structure can thus produce temporary maladaptation to the novel environment and cause the approach to the optimum ecotype in the new environment to be greatly slowed [32, 64]. There may, for example, be a negative correlation between thermal tolerance and survivorship or other fitness related traits, under the novel environmental conditions [16]. Individuals with a high tolerance to extreme cold may also have higher maintenance energy requirements that could negatively influence growth and survival in environments characterized by low temperatures [13].

Our results only partially support the Climate Variability Hypothesis [1], which states that greater thermal tolerance breadths and greater beneficial thermal acclimation abilities in high latitude populations or species (compared to tropical ones) may have arisen as adaptations in order to cope with increased climatic variability towards the poles [1]. While we do find that thermal tolerance breadths are improved following adaptation to more variable climates, we also observe that the evolved increase in tolerance breadth is actually not observed in the evolved climate, thus negating any matching of thermal breadth to the breath of experienced environmental conditions. Moreover, in contrast to the Climatic Variability Hypothesis, we find that increased climatic variability results in maladaptive thermal plasticity, rather than adaptive thermal plasticity, at least during the initial stages of adaptation. Greater thermal tolerance breadth at high latitudes may instead reflect historical processes associated with the range shift and the spatial variation in selection history of an increasingly more variable environment faced by range-shifting populations [1, 37, 56]. The degree of thermal change simulated in this experiment approximates changes expected by a poleward shifting organism, which has opportunities to disperse each year. It is likely for many species that the rate of thermal change experienced in the wild is likely to vary from what we present here (2 °C decrease per generation).

Our results invoke the possibility that maladaptive plasticity may be a chronic component of adaptation to progressively more stressful conditions (such as during a range shift or during periods of ongoing, intergenerational environmental change), because as populations are adapting, they are also progressively encountering greater levels of environmental stress. Other studies have found similar evidence for maladaptive plasticity in different traits in the first stages of adaptation. Ghalambor et al., [18] found Trinidadian guppies initially exhibited maladaptive plasticity in brain gene expression patterns when transplanted to a novel, predator free environment, because selection has not yet had an opportunity to act on the genetic variation for plasticity. Moreover, recent modelling work has suggested that maladaptive plasticity, when it arises in the context of adaptation along an environmental gradient, may potentiate adaptation by increasing additive genetic variance and thus the efficacy of selection to facilitate genetic divergence between populations connected by gene flow. Additionally, it increases the distance that genetic change has to cover to reach the new optimum leading to a stronger response to selection [53]. However, in the case where maladaptive plasticity results in countergradient variation, it also minimizes the range of phenotypes (in this case, thermal tolerance) produced along an environmental gradient. Countergradient variation can thus reduce tolerance breadth and thus ability of populations to sustain further environmental change, in comparison to other forms of adaptive evolution [8, 53].

## Conclusions

We propose that the ordering of thermal adaptation during a range expansion may involve genetic compensation arising in the initial stages of adaptation, preceding the later evolution of adaptive thermal plasticity as environments become more predictable [37, 38]. Temporal, evolutionary shifts from primarily maladaptive to adaptive plasticity as a range shift progresses implies that in the initial stages of adaptation to the novel climate in their new range, species’ range expansion trajectories may be at their most vulnerable, because they have not yet evolved the adaptive plasticity required to anticipate novel thermal conditions beyond their current range front [29]. In the context of global pest or disease species, this may have important implications for a time limit on our ability to control populations establishing in a novel environment. Rapid eradication may be critical, when maladaptive plasticity is still present and before the acquisition of beneficial developmental plasticity which may ultimately allow a range expansion to gain momentum.

## Supplementary information

**Additional file 1.** Supplementary Information.

**Additional file 2.***Callosobruchus maculatus* Thermal Tolerance Raw Aata.

## Data Availability

All data generated or analysed during this study are included in this published article [and its Additional file 2].

## Abbreviations

C: constant conditions
CT_max_: critical thermal maximum
CT_min_: critical thermal min
E + D + A: evolution, developmental plasticity, and acclimation treatment
E + D: evolution and developmental plasticity treatment
E: evolution and developmental plasticity treatment
