# Supplementary material for: Maladaptive plasticity facilitates evolution of thermal tolerance during an experimental range shift
Source: BMC Evol Biol. 2020 Apr 23;20:47. doi: 10.1186/s12862-020-1589-7 (PMC7181507; doi:10.1186/s12862-020-1589-7)
Supplement: Supplementary file 1 — Additional file 1. Supplementary Information. [file 12862_2020_1589_MOESM1_ESM.docx]

# **Maladaptive plasticity facilitates evolution of thermal tolerance during an experimental range shift.**

Supporting Information

Supplementary Results

Table S1, S2

## Maximum potential effect of experimental replicate line on the reported results

Table S1: Individuals from each replicate evolved line and developmental treatment were balanced across acclimation treatments, but information on replicate ID was lost during thermal performance trials. To assess for possible effects of evolved replicate on our results, we designated a new variable which describes the maximum amount of variation that could be explained by the replicate line from which individuals were obtained. We did this by consistently assigning individuals with the lowest thermal tolerance within each treatment to a psuedo ‘replicate 1’, on up to individuals with the highest tolerances within each treatment category being assigned ‘replicate 5’. Including this variable as a random effect in mixed models (using lme4 and lmerTest models for R) (Bates et al. 2011 and Kuznetsova et al. 2018) indicates that replicate could explain as much as 71-79% of the variation in thermal tolerance, if replicate were perfectly correlated with thermal tolerance. However, estimates and significance levels of the fixed effect of treatment are unaffected by the inclusion of this pseudo-replicate random effect, suggesting that the fixed effects of temperature are robust, after accounting for any possible level of lineage-level variation in response rate

| Lines | Treatment | Cold Tolerance | Estimate | SE | Pr(>\|t\|) | Heat Tolerance | Estimate | SE | Pr(>\|t\|) |
| --- | --- | --- | --- | --- | --- | --- | --- | --- | --- |
| Control | C | Baseline |  |  |  | Baseline |  |  |  |
| Fluctuating | E | Improved | -3.98 | 0.4393 | 4.44e-16 | Improved | 0.42 | 0.1058 | 8.39e-05 |
|  | E+D | Same | -0.7650 | 0.4393 | 0.08362 | Improved | 0.5725 | 0.1058 | 2.36e-07 |
|  | E+D+A | Same | 0.3307 | 0.4393 | 0.45265 | Worse | -0.5950 | 0.1058 | 8.63e-08 |
| Constant  Decline | E | Same | -0.095 | 0.3756 | 0.801 | Improved | 0.3100 | 0.1318 | 0.020 |
|  | E+D | Improved | -2.0025 | 0.3756 | 3.45e-07 | Same | -0.0950 | 0.1318 | 0.472 |
|  | E+D+A | Same | -2.9825 | 0.3756 | 4.12e-13 | Worse | -0.5613 | 0.1318 | 3.61e-05 |

Table S2: Post-hoc statistical comparisons of thermal tolerances among evolved, developmental and acclimation treatment regimes. E = Evolved regime compared to control (E compared to C; as in table 1), E+D = additional effect of developmental regime, over evolved regime (E+D compared to E), and E+D+A = effect of short term acclimation, on top of developmental regime (E+D+A compared to E+D), E+D+A = effect of short term acclimation and developmental regime combined compared to evolved regime (E+D+A compared to E). After Bonferroni correction, ∝ for statistical significance = 0.0167.

| Lines | Treatment | Comparison | Cold Tolerance | Estimate | SE | Pr(>\|t\|) | Heat Tolerance | Estimate | SE | Pr(>\|t\|) |
| --- | --- | --- | --- | --- | --- | --- | --- | --- | --- | --- |
| Fluctuating | E+D | E | Worse | 3.2150 | 0.7815 | 7.27e-05 *** | Same | 0.1450 | 0.1859 | 0.437 |
|  | E+D+A | E+D | Same | 1.0958 | 0.7815 | 0.164 | Worse | -1.1675 | 0.1859 | 5.99e-09 *** |
|  | E+D+A | E | Worse | 4.3108 | 0.7815 | 2.11e-07 *** | Worse | -1.0225 | 0.1859 | 2.27e-07 *** |
| Constant  Decline | E+D | E | Improved | -1.9075 | 0.5565 | 0.00084*** | Same | -0.4050 | 0.2072 | 0.053 |
|  | E+D+A | E+D | Same | -0.9800 | 0.5565 | 0.08083 | Worse | -0.4662 | 0.2072 | 0.0263 |
|  | E+D+A | E | Improved | -2.8875 | 0.5565 | 8.99e-07 *** | Worse | 0.8712 | 0.2072 | 5.13e-05 *** |
